# Superconductivity in Te-Deficient ZrTe_2_

**DOI:** 10.1021/acs.jpcc.2c08836

**Published:** 2023-03-08

**Authors:** Lucas E. Correa, Pedro P. Ferreira, Leandro R. de Faria, Vitor M. Fim, Mario S. da Luz, Milton S. Torikachvili, Christoph Heil, Luiz T. F. Eleno, Antonio J. S. Machado

**Affiliations:** † Universidade de São Paulo, Escola de Engenharia de Lorena, DEMAR, 12612-550 Lorena, Brazil; ‡ Institute of Theoretical and Computational Physics, Graz University of Technology, NAWI Graz, 8010 Graz, Austria; ¶ Instituto de Ciências Tecnológicas e Exatas, Universidade Federal do Triângulo Mineiro, 38025-180 Uberaba, Minas Gerais, Brazil; § Department of Physics, San Diego State University, San Diego, California 92182-1233, United States

## Abstract

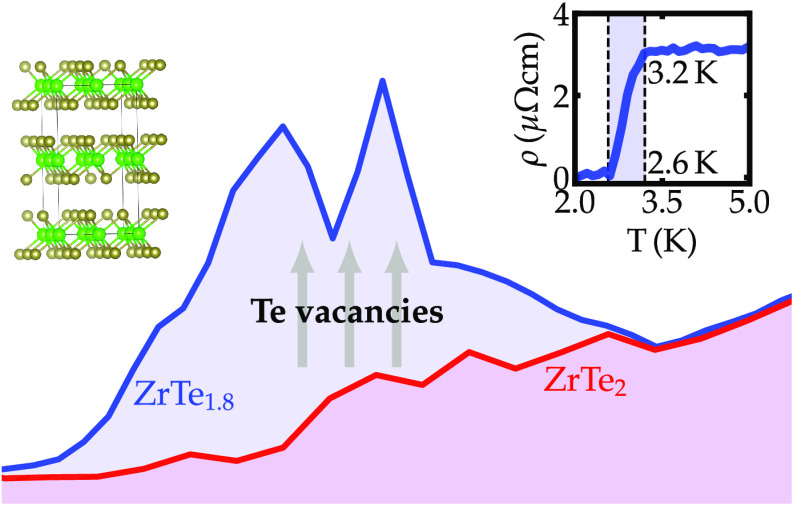

We present structural, electrical, and thermoelectric potential measurements on high-quality single crystals of ZrTe_1.8_ grown from isothermal chemical vapor transport. These measurements show that the Te-deficient ZrTe_1.8_, which forms the same structure as the nonsuperconducting ZrTe_2_, is superconducting below 3.2 K. The temperature dependence of the upper critical field (H_*c*2_) deviates from the behavior expected in conventional single-band superconductors, being best described by an electron–phonon two-gap superconducting model with strong intraband coupling. For the ZrTe_1.8_ single crystals, the Seebeck potential measurements suggest that the charge carriers are predominantly negative, in agreement with the ab initio calculations. Through first-principles calculations within DFT, we show that the slight reduction of Te occupancy in ZrTe_2_ unexpectedly gives origin to density of states peaks at the Fermi level due to the formation of localized Zr-*d* bands, possibly promoting electronic instabilities at the Fermi level and an increase at the critical temperature according to the standard BCS theory. These findings highlight that the Te deficiency promotes the electronic conditions for the stability of the superconducting ground state, suggesting that defects can fine-tune the electronic structure to support superconductivity.

## Introduction

The transition metal dichalcogenides (TMDs), with chemical composition TX_2_, where T = Zr, Hf, Ti, Mo, W, Ta, etc., and X = S, Se, Te, display a rich number of important physical properties and have the potential for many applications.^1−11^ These materials frequently crystallize in low-dimensional structures and exhibit coherent states and electronic instabilities, such as charge density waves (CDW) and superconductivity (SC).^12−22^ Their low-dimensional, layered structures are held together by weak van der Waals forces, and SC can emerge upon intercalation of different species in the interstices of the structural van der Waals gap.^23−26^ In addition to SC and CDW, recent studies suggest that many of these TMDs exhibit nontrivial topology, especially type-II topological Dirac states, increasing the interest in this class of materials.^27−31^

The focus of this work is the ZrTe_2–*x*_ TMD, a Te-deficient compositional modification of the ZrTe_2_ which crystallizes in the well-known CdI_2_-prototype structure *P*3̅*m*1 (164),^32^ which is attracting intense attention from the physical chemistry research community.^33−43^ Ionic intercalation can be accomplished in ZrTe_2_, by positioning the foreign species in the van der Waals gap, frequently leading to SC, e.g., Cu_*x*_ZrTe_2_ (*T*_*c*_ ≈ 9.0 K) and Ni_*x*_ZrTe_2_ (*T*_*c*_ ≈ 4.0 K).^44,45^ In the case of Ni_*x*_ZrTe_2_, for instance, the possible coexistence of multigap superconductivity and CDW (*T*_CDW_ ≈ 287 K) instabilities are well established. Additionally, recent angle-resolved photoemission spectroscopy and de Haas–van Alphen oscillations experiments also suggest that ZrTe_2_ can be regarded as a Dirac semimetal with massless 4-fold quasiparticles.^46,47^ Due to the possible coexistence of SC and nontrivial topological properties, ZrTe_2_ is an excellent candidate for probing the interplay between different emergent quantum states.

While the connection between intercalation and SC has been reported in ZrTe_2_,^44,45^ the effect of slight compositional variations and defects has not been probed. Here we address the effect of Te deficiency on the electronic properties of ZrTe_2_, and we show that SC can emerge due to slight structural and electronic modifications. Vacancies at the Te sites are introduced in a controlled fashion using isothermal chemical vapor transport (ICVT) growth, and the emergence of SC is characterized by means of electrical resistivity, AC susceptibility, and thermoelectric potential measurements. Our measurements suggest that ZrTe_1.8_ is a two-gap superconductor below approximately 3.2 K with relatively strong intraband coupling. Furthermore, we calculated the band structure of ZrTe_1.75_ within the density functional theory and supercell method. We found that the inclusion of vacancies in bulk ZrTe_2_ gives rise to a peak at the density of states (DOS) at the Fermi level due to the formation of localized Zr-*d* bands, bridging the way to electronic instabilities at the Fermi surface.

## Methods

### Experimental Details

The ZrTe_2–*x*_ single crystals were prepared by means of isothermal chemical vapor transport (ICVT), recently proposed by some of the present authors.^48^ Prereacted ZrTe_2–*x*_ pellets were synthesized by a solid-state reaction, and they served as precursors for the ICVT growth. The pellets and a small amount of iodine, which serves as a transport agent, were sealed in a quartz tube, and the growth took place over 7 days at temperatures of 950 °C. As a result, the crystals grow out of the pellets, and the typical dimensions of the largest crystals are 10 × 10 × 0.1 mm^3^. The growth details are discussed in ref (48).

The composition was determined from energy-dispersive spectroscopy (EDS) and induced coupling plasma (ICP) utilizing microwave plasma atomic emission spectroscopy (MP-AES) after dilution in HNO_3_ and HCl. For the MP-AES measurements, sample replicates, reagent blanks, and standard samples with precisely known compositions were used for cross-checking and ensuring accuracy. The crystallographic quality of crystals was verified by X-ray diffraction (XRD) using a Panalytical–Empyrean diffractometer. Rocking curves centered on the (00*l*) reflections were used to ascertain the orientation and level of crystallinity.

The electrical resistivity, AC susceptibility, and thermoelectric potential measurements were carried out with the Physical Property Measurement System PPMS-9 from Quantum Design, equipped with a 9.0 T superconducting magnet. For the 4-probe resistivity measurements, four copper leads were attached to the sample using silver paste. The typical contact resistance was in the 4–5 Ω range. The magnetization measurements were performed using the vibration sample magnetometer (VSM). The AC susceptibility measurements were carried out with the ACMS II option of the PPMS, with excitation fields of 1 and 2 Oe, in the frequency range from 1000 to 4000 Hz. The Seebeck coefficient was measured using the thermal transport option of the PPMS-9. The sample was placed across a small printed circuit board section containing four copper lines. Contact of the sample with the copper lines was established with Ni-loaded epoxy.

### Computational Methods

First-principles electronic-structure calculations were performed within the Kohn–Sham scheme^49^ of the Density Functional Theory (DFT)^50^ with scalar-relativistic optimized norm-conserving Vanderbilt pseudopotentials^51^ as implemented in Quantum Espresso.^52,53^ Exchange and Correlation (XC) effects were treated with the generalized gradient approximation (GGA) according to vdW-DF2-C09 parametrization,^54,55^ explicitly including the nonlocal van der Waals interactions. All numerical parameters were exhaustively tested to guarantee a total energy convergence lower than 3 meV/atom. Based on the convergence results, we adopted a kinetic energy cutoff of 60 Ry for the wave functions and 240 Ry for the charge density and potential and a centered 4 × 4 × 4 *k*-point sampling in the first Brillouin according to the Monkhorst–Pack scheme.^56^ A denser 24 × 24 × 24 *k*-point grid was used to obtain the DOS. Self-consistent-field calculations were carried out using the Methfessel–Paxton smearing^57^ with a spreading of 0.005 Ry for Brillouin-zone integration, while the optimized tetrahedron method^58^ was adopted for the electronic occupation in non-self-consistent-field calculations. All lattice parameters and internal degrees of freedom were relaxed to achieve a ground-state convergence of 10^–7^ Ry in total energy and 10^–6^ Ry/*a*_0_ for forces acting on the nuclei. The convergence criteria for self-consistency adopted was 10^–10^ Ry. The supercells were generated with the supercell code.^59^

## Results and Discussion

**Figure 1:**
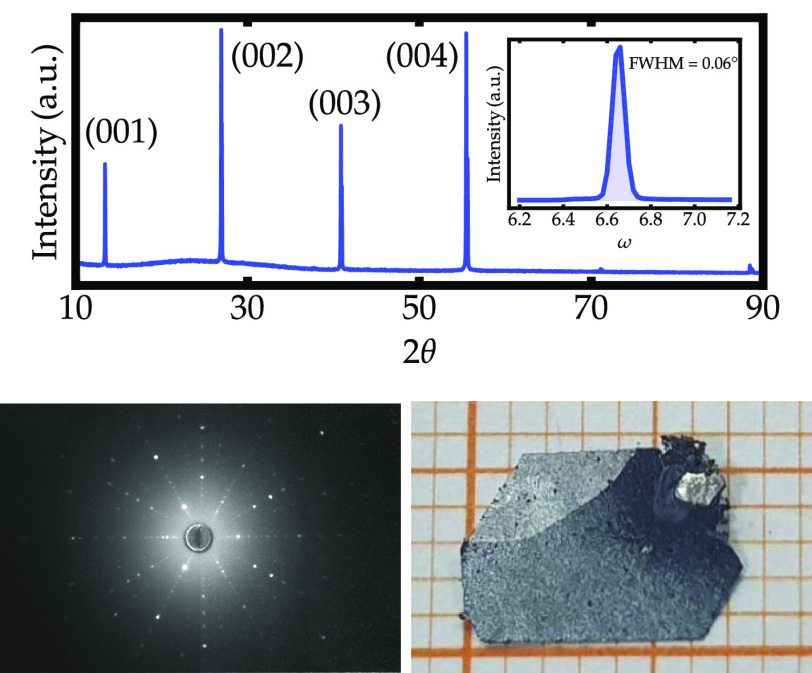
θ–2θ XRD scan of the ZrTe_1.8_ crystal with beam incident on the flat surface (*ab*-plane). The inset shows the rocking curve centered on the (001) reflection. The lower panel shows the Laue patterns of the ZrTe_1.8_ reciprocal lattice (left) and the picture of one representative single crystal with dimensions 6.5 × 5.5 × 0.1 mm^3^ (right).

We synthesized single crystals from a precursor with nominal composition ZrTe_1.8_. The EDS and ICP elemental analysis indicated ZrTe_1.8_ and ZrTe_1.85_ compositions, respectively. For simplicity, heretofore, we will refer to the ZrTe_1.8_ composition. A θ–2θ XRD scan with the incident beam on the flat surface of a crystal (*ab*-plane) is displayed in Figure 1. It only shows (00*l*) reflections, which suggests that flat faces are the basal plane of the trigonal CdI_2_ structure. The XRD scan is consistent with the Zr–Te phase diagram,^60^ where the *P*3̅*m*1 space group was experimentally determined to be stable in a wide composition range, i.e., ZrTe_2–*x*_ (*x* = 0 to −0.28). The inset of Figure 1 shows the rocking curve centered on the (001) reflection. This reflection is centered at θ = 6.66°, and the full width at half-maximum (fwhm) is ∼0.06°. The narrow fwhm is consistent with excellent crystallinity. The Te-deficient crystals had a dull silvery appearance and typical sizes of 6 × 6 × 0.05 mm^3^, as shown in Figure 1.

**Figure 2:**
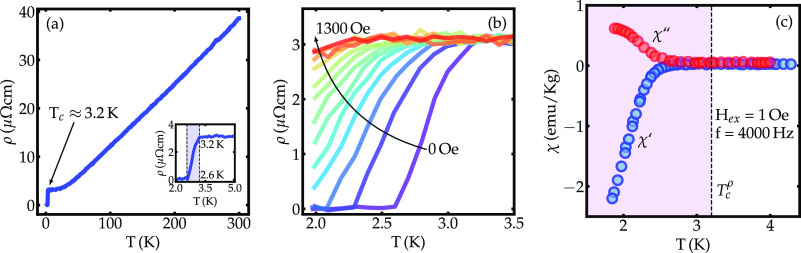
(a) Temperature dependence of the electrical resistivity for the ZrTe_1.8_. The inset presents the low-temperature region, showing the onset of the SC transition near 3.2 K and the completion at 2.6 K. (b) Effect of magnetic fields up to 1300 Oe on the resistive transition to SC. (c) AC magnetic susceptibility χ versus temperature for an applied magnetic field of 1 Oe and frequency 4000 Hz.

The temperature dependence of the electrical resistivity of the ZrTe_1.8_ is shown in Figure 2a. The resistivity drops nearly linearly upon cooling from 300 K, it starts leveling off around 40 K, and eventually drops to zero with the onset of SC at *T*_*c*_ ≈ 3.2 K. Figure 2b shows the effect of the magnetic field on the resistive transition to SC in fields up to 1300 Oe. The shift of the 3.2 K transition to lower temperature as a function of the applied magnetic field is consistent with SC. Further support for bulk superconductivity is provided by the AC magnetic susceptibility χ near *T*_*c*_, as shown in Figure 2c, for data collected with an excitation field H_*ex*_ = 1 Oe and frequency *f* = 4000 Hz. While in-phase component χ′ reveals a large diamagnetic signal below *T*_*c*_, the out-of-phase component χ^″^ increases, reflecting the dissipation associated with the onset of flux dynamics.

**Figure 3:**
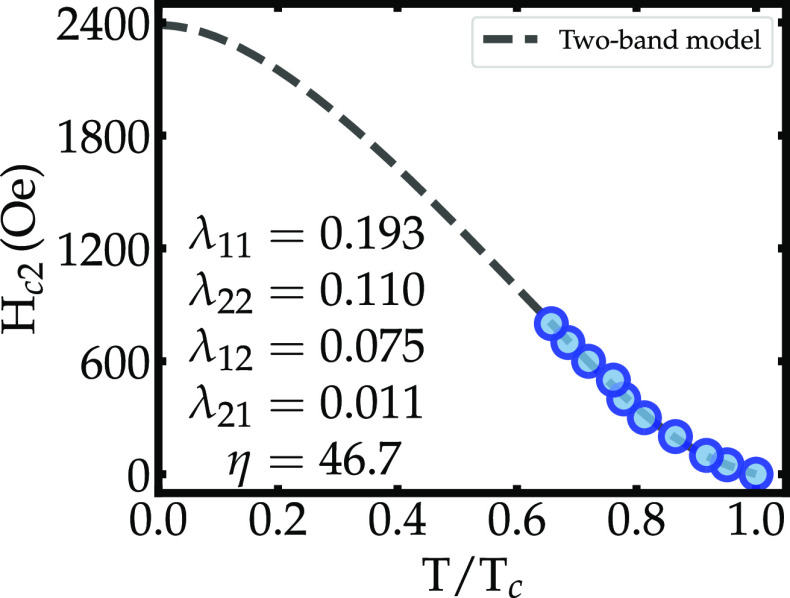
Upper critical magnetic field H_*c*2_ vs reduced temperature *T*/*T*_*c*_. The values of H_*c*2_ were taken from the onset of the resistive transitions. The dashed line is a fit to the data using Gurevich’s two-band model.

Using the magneto-resistance data of Figure 2 and taking *T*_*c*_ from the onset of the superconducting transitions, the upper critical field H_*c*2_ can be plotted as a function of the reduced temperature *t* = *T*/*T*_*c*_, as shown in Figure 3.

The H_*c*2_(*T*) data show an upturn below *T*_*c*_, in sharp contrast with the expected quadratic behavior of single-band superconductivity and the Werthamer–Helfand–Hohenberg (WHH) single-gap model.^61^ The positive curvature of H_*c*2_ below *T*_*c*_ is frequently taken as an indication of multiband SC.^62−67^ Alternatively, the fit of the H_*c*2_(*T*) data to the two-band model proposed by Gurevich^68^ results in an excellent fit, as seen in Figure 3, where it is represented by the dashed black line. In Gurevich’s model,^68^ the equation for H_*c*2_ takes the form
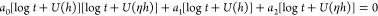
1where *a*_0_ = 2*w*/λ_0_, *a*_1_ = 1 + λ_–_/λ_0_, and *a*_2_ = 1 – λ_–_/λ_0_, with λ_–_ = λ_11_ – λ_22_, *w* = λ_11_λ_22_ – λ_12_λ_21_, and . The coefficients λ_*mn*_ are the eigenvalues of the BCS superconducting coupling matrix. The diagonal terms λ_11_ and λ_22_ quantify the intraband coupling, whereas the off-diagonal terms λ_12_ and λ_21_ describe the interband coupling. The function *U*(*x*) is defined as *U*(*x*) = ψ(1/2 + *x*) – ψ(1/2), where ψ(*x*) is the digamma function. The arguments of the *U*(*x*) in eq 1 are given by *h* = *H*_*c*2_*D*_1_/2ϕ_0_*T* and η = *D*_1_/*D*_2_, where *D*_1_ and *D*_2_ are the intraband electronic diffusivities of bands 1 and 2 in the normal state and ϕ_0_ is the magnetic flux quanta. The derivation of the intraband diffusivity tensors *D*_*m*_^*αβ*^ expressed in terms of microscopic parameters can be found in Appendix A of ref (68).

The fit to the two-band model yields a H_*c*2_(0) value of ∼2400 Oe, which is consistent with the trend from the H_*c*2_ data extracted from the resistivity measurements. The diffusivity and coupling parameters yielded by the two-band fit are λ_11_ = 0.193 and λ_22_ = 0.110 (intraband coupling), λ_12_ = 0.075 and λ_21_ = 0.011 (interband coupling), and η = 46.7. The high diffusivity ratio η reflects the significant difference between the electron mobility of distinct Fermi Surface sheets involved in the pairing mechanism, which leads to the positive curvature of H_*c*2_(*T*). Still, the λ_*mn*_ values extracted from the effective model suggest that the intraband coupling is 1 order of magnitude higher than the interband scattering, which likely is the main driving force for the multiband-type behavior observed. Consistently, SC in ZrTe_2_ intercalated with Ni and Cu has also been linked to multiband behavior.^44,45^ Given that SC in Te-deficient ZrTe_1.8_ is also consistent with multiband behavior, we propose that the multigap SC state often observed in intercalated TMDs is not necessarily related to the intercalation, but rather it is intrinsically related to the TMDs electronic structure.

**Figure 4:**
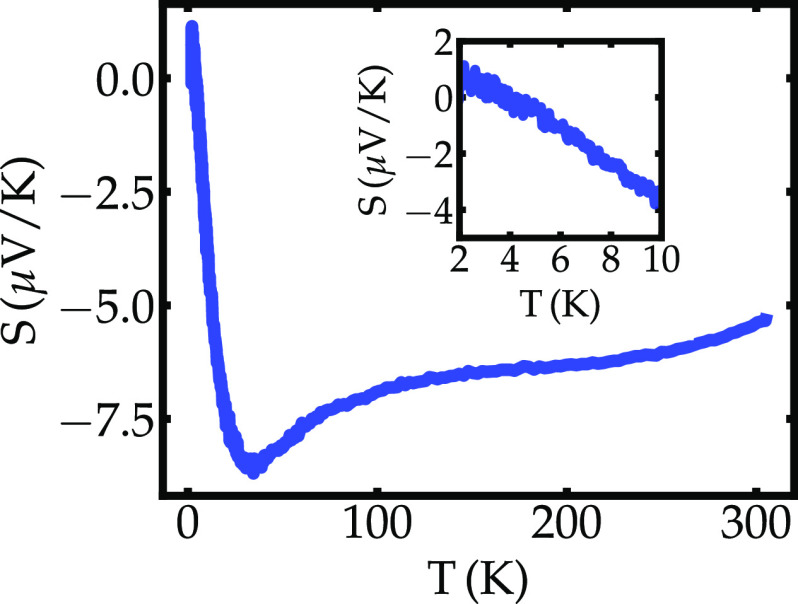
Seebeck coefficient as a function of temperature for ZrTe_1.8_.

Measurements of the thermoelectric potential (Figure 4) suggest that the preponderance of charge carriers are electrons. The Seebeck potential is ∼−5.2 μV/K near ambient temperature. It becomes slightly more negative upon cooling, reaching a minimum (∼−8.5 μV/K) near 35 K, and increasing rapidly below 20 K, reaching zero value near *T*_*c*_, consistent with SC pairing. The complex behavior of *S*(*T*) near 35 K possibly results from the convoluted interplay between the temperature dependence of the electron concentration, the asymmetry of the electron distribution near the Fermi level, the mean free path, and mean scattering time. To the best of our knowledge, this is the first time that bulk SC was observed in ZrTe_2_, albeit Te-deficient, without intercalation or pressure. The emergence of SC in Te-deficient ZrTe_2_ suggests that the Te vacancies or the resulting crystalline defects play a crucial role, and a better understanding is still in order.

To probe the effects of tellurium defects, we generated 2 × 2 × 2 supercells by occupying 85% of the two nonequivalent Te atomic sites within the 2*d* Wyckoff position of the unit cell. With that, there are 64 possible structural configurations, which we can reduce to only four unique clusters (with different multiplicities) by identifying and employing the full symmetry operations of each supercell.

**Figure 5:**
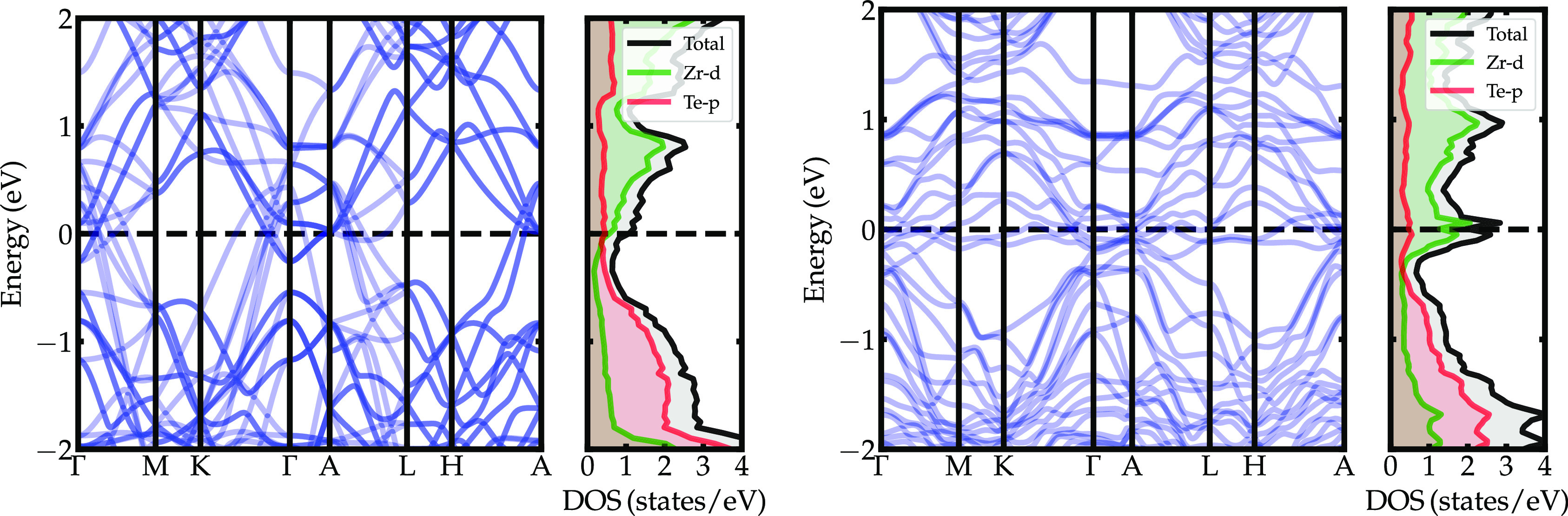
(Left) Electronic band structure and DOS for pure ZrTe_2_ in a 2 × 2 × 2 supercell. (Right) Electronic band structure for the cluster with the lowest formation energy and averaged DOS for ZrTe_1.75_ in a 2 × 2 × 2 supercell. The averaged DOS was weighted according to the degeneracy of each nonequivalent structural configuration.

Figure 5 shows the electronic structure for the pure 2 × 2 × 2 ZrTe_2_ supercell and the averaged Te-deficient supercell with composition ZrTe_1.75_ weighted according to the cluster degeneracies. The total DOS at the Fermi level for pure ZrTe_2_ is 1.1 states/eV. This value is consistent with previous calculations, which include the spin–orbit coupling,^45^ yielding 1.0 states/eV at the Fermi level, a percentage difference of only 8% with respect to our results without including spin–orbit coupling. From the 1.1 states/eV, approximately 53% originate from the Zr-*d* electron pockets, whereas 42% are coming from the Te-p hole pockets. With the inclusion of Te vacancies, a narrow DOS peak at the Fermi level develops due to the formation of localized, near-flat Zr-*d* electronic bands along the entire extent of the Brillouin zone. The averaged ZrTe_1.75_ has 2.1 states/eV at the Fermi level, an increase of 92% compared to pure ZrTe_2_, of which approximately 65% are derived from the Zr-*d* orbitals, and only 23% are coming from the Te-*p* orbitals. Since most of the Zr-*d* states are electron-like pockets at the Fermi surface, the increase of the Zr-*d* character at the Fermi level is consistent with the polarity of the Seeback potential determined experimentally, revealing that the charge carriers are predominantly negative.

According to the BCS theory, the electronic states that contribute the most to SC are those with energies within a range of the order of *ℏω*_*D*_ around the Fermi energy, where ω_*D*_ is the Debye frequency, and, assuming that the DOS within the energy range *ℏω*_*D*_ is constant, the superconducting critical temperature follows the relation *T*_*c*_ ≈ *ℏω*_*D*_ exp[−1/*N*(ϵ)λ], where λ is the electron–phonon coupling strength.^69^ Therefore, the increased DOS at the Fermi level due to the formation of Te vacancies leads to a significant increase in the critical temperature, explaining the relatively high critical temperature in ZrTe_1.8_ compared to the non-superconducting defect-free compound. Furthermore, the Te vacancies also give rise to a sharp DOS peak at *E*_*F*_, similar to van Hove-type singularities; i.e., the DOS varies rapidly within the *ℏω*_*D*_ energy range. When electron correlation effects are considered, this logarithmic instability also enhances *T*_*c*_ and favors spontaneous symmetry-breaking phase transitions.^70−75^ Therefore, the appearance of localized states near the Fermi level and the substantial increase of the total DOS at *E*_*F*_ reasonably explains on qualitative grounds the defect-induced SC in ZrTe_2_.

In order to describe the superconducting state observed in ZrTe_1.8_, one would need to employ advanced techniques such as the fully anisotropic Migdal–Eliashberg theory^76^ or Superconducting Density Functional Theory^77−79^ to accurately take into account the anisotropy of the electron–phonon coupling and the two-gap feature of the superconducting gap function. As the presence of Te vacancies necessitates the consideration of supercells and very dense samplings of the Brillouin zone are required to obtain convergence, such a calculation is beyond the scope of the current work but will be the focus of a future project.

## Conclusions

This work shows the emergence of superconductivity in ZrTe_1.8_, a Te-deficient, off-stoichiometry composition of the nonsuperconducting TMD ZrTe_2_, on high-quality single crystals synthesized by ICVT. The superconducting properties were characterized by measurements of electrical resistivity, AC susceptibility, and thermoelectric potential. The ZrTe_1.8_ composition gives rise to a multigap superconducting state with a critical temperature close to 3.2 K. Interestingly, the presence of a DOS peak at the Fermi level due to localized Zr-*d* bands can be linked to the superconducting pairing in the Te-deficient ZrTe_2_. These results strongly suggest that native point defects, such as vacancies, are essential for SC in the widely investigated class of transition-metal dichalcogenides. Furthermore, we show that the multiband nature is intrinsic to ZrTe_2_ and that these findings are possibly extending to the whole family of TMDs.
